# Oral health behavior of children and guardians’ beliefs about children’s dental caries in Vientiane, Lao People’s Democratic Republic (Lao PDR)

**DOI:** 10.1371/journal.pone.0211257

**Published:** 2019-01-25

**Authors:** Somphone Phanthavong, Daisuke Nonaka, Thongsavanh Phonaphone, Kyoko Kanda, Phouphachanh Sombouaphan, Norie Wake, Sangvane Sayavong, Toshiyuki Nakasone, Khampe Phongsavath, Akira Arasaki

**Affiliations:** 1 Dental Division, Setthathirath Hospital, Vientiane, Lao PDR; 2 Faculty of Dentistry, University of Health Sciences, Vientiane, Lao PDR; 3 School of Health Sciences, Faculty of Medicine, University of the Ryukyus, Okinawa, Japan; 4 International Collaboration Section, General Strategic Planning Division, University of the Ryukyus, Okinawa, Japan; 5 Department of Oral and Maxillofacial Functional Rehabilitation, Graduate School of Medicine, Okinawa, University of the Ryukyus, Japan; College of Medicine, National Taiwan University, TAIWAN

## Abstract

Dental caries is considered a major health problem among schoolchildren in Lao People’s Democratic Republic (Lao PDR). According to Health Belief Model (HBM)-based research, children’s oral health behavior can be determined by their guardians’ beliefs. This study aimed to describe children’s oral health behavior and its association with childhood dental caries, as well as to assess associations between children’s tooth-brushing behavior and guardians’ beliefs in an urban area of Lao PDR, using HBM. Data were collected from ten primary schools in the Sisattanak district, the Vientiane capital, between 2013 and 2014. Ten dentists with the help of dental hygienists and schoolteachers conducted dental health check-ups at the schools that diagnosed dental caries based on visual inspection. They also conducted a questionnaire-based survey with the schoolchildren’s guardians to collect data including socio-economic and demographic information, their children’s oral health behavior, and guardians’ beliefs derived from HBM, including perceived susceptibility to and perceived severity of child dental caries, perceived benefit of and perceived barrier to child’s tooth brushing, and self-efficacy in making their children brush their teeth twice daily. A mixed-effects logistic regression model assessed the association between dental caries and children’s oral health behavior and between children’s tooth-brushing behavior and guardians’ beliefs. Data from 1161 of 1304 (89.0%) children registered at the schools were used. The prevalence of dental caries was 82%. Children who brushed their teeth ≥ twice/day were significantly less likely to have dental caries than those brushing once or seldom (OR: 0.64, 95% CI: 0.45 to 0.91). The number of children who brushed twice daily also significantly increased with the increased level of guardians’ self-efficacy (OR: 2.14, 95% CI: 1.91 to 2.41). In conclusion, childhood dental caries was associated with daily tooth brushing. Children’s tooth-brushing behavior was associated with guardians’ self-efficacy in making their children brush twice daily.

## Introduction

The Lao People’s Democratic Republic (Lao PDR) is a lower-middle-income country situated in Southeast Asia. Among Lao schoolchildren, dental caries was associated with impairments of daily life activities, such as eating and sleeping, and toothache was one of the major reasons for school absenteeism [1, 2]. The National School Health Policy of the Lao PDR, which was established in 2006, includes dental caries as one of the target diseases that should be addressed through school-based strategies. Promoting appropriate oral health behavior is key to preventing dental caries. According to the World Health Organization [3], primary schoolchildren should be educated to be able to 1) practice proper oral hygiene care, 2) restrict the amount and frequency of sugar intake, and 3) adopt a regular check-up routine. In Lao PDR, however, little has been reported about how common schoolchildren practice these health behaviors and what the determinants of these behaviors actually are.

A Cochrane systematic review on school-based behavioral interventions for preventing childhood dental caries concluded that there is a need for research to utilize theory in the design and evaluation of interventions for improving children’s oral health behaviors [4]. Additionally, the World Health Organization suggested that the role of parents is critical in shaping their children’s positive attitudes and promoting their children’s oral healthy behaviors at home and, thus, parents should be involved in planning and implementing a program that aims at oral health education and promotion for their children [3].

In Lao PDR, the Fit for School program promoted a daily tooth brushing and hand hygiene at selected primary schools and demonstrated the effectiveness of daily tooth brushing with fluoride toothpaste at school in reducing increment of dental caries [5]. However, it remains poorly understood how such school-based intervention programs can effectively involve parents in planning and implementing the program while utilizing health theories.

The Health Belief Model (HBM) is one of the commonly used health theories for oral health behavior [6–8]. HBM is comprised of five beliefs as theoretical constructs: perceived severity of a disease, perceived susceptibility to a disease, perceived benefit of adopting the behavior, perceived barrier against adopting the behavior, and self-efficacy in adopting the behavior [7]. Multiple studies have shown significant effects of parental beliefs on the teeth brushing behavior of their children [9–12]. For example, a study conducted with parents of young children in the USA reported that parents who brushed their children’s teeth less than twice daily were more likely to hold a false belief about the benefit of twice daily tooth-brushing (i.e., perceived benefit) [9]. An international study conducted in 17 countries showed parental self-efficacy in engaging in twice-daily tooth brushing of their children to be associated with twice daily brushing by their children aged between 3 to 4 years [10]. Similar findings were reported elsewhere [11,12]. For young children, evidence has been accumulating on the importance of the parental beliefs about the tooth-brushing behavior of their children; i.e., beliefs about the control ability, benefit, and barrier of their children’s tooth-brushing behavior and beliefs about the susceptibility to and severity of childhood dental caries. For children of school-going age, however, the evidence is extremely limited.

Therefore, this study aimed to describe children’s oral health behavior and its association with childhood dental caries, as well as to assess associations between children’s tooth-brushing behavior and guardian’s beliefs in an urban area of Lao PDR, using HBM.

## Materials and methods

### Study site and population

The present study used data that were collected for the *Cha-ganjyu* (long-life) School and Community Based Oral Health Promotion Project. Setthathirath Hospital, Lao PDR, and the University of the Ryukyus, Japan, started the project at 10 of the 20 public primary schools in Sissathanack district, Vientiane capital, in 2012. There were three school clusters of the public primary schools in the district. The project purposively included three or four schools from each cluster, expecting that the included schools would share their experience of the project with the non-included schools within school cluster. The Sissathanack District Education Office selected the 10 schools for the project, considering the conditions of the schools; e.g., the presence/absence of a similar project at school and the willingness of the school principal to participate in the project. The major activities of the project included teacher-supervised daily tooth brushing by children at school, weekly fluoride rinsing at school, bi-annual dental check-ups at school, and dental treatment with the first-grade children at Setthathirath Hospital. The total number of children registered from the first to fifth grade in the 10 schools was 1304, and the data of 1161 children (89.0%) were included in the present study.

### Data collection

Ten dentists with the help of dental hygienists and schoolteachers conducted a dental health check-up of the schoolchildren of all 10 schools in October 2013 and collected data on dental caries. They also conducted a questionnaire-based survey with the guardians of the schoolchildren in September 2014. The guardians were requested to gather at respective schools on a designated day and were invited to the questionnaire-based survey. The guardians responded to a questionnaire (S1 File) at school, with the help of researchers. The data collected by the questionnaire-based survey included socio-economic and demographic information, their beliefs that were derived from the HBM, and the oral health behavior of their children. First, the authors developed the questionnaire in English, referring to HBM-based studies [13,14]. This was then translated into Lao language and pre-tested with the guardians whose children went to a non-study primary school in Sissathanack district.

### Variables

#### Dental caries

The dental examination performed during the dental check-up to determine the presence/absence of dental caries followed the methods and criteria described by the World Health Organization [15]. The dental examination was conducted in respective schools under natural daylight, using a plane mouth mirror and periodontal probe. Dental caries experience was recorded using the DMFT/dmft index. No calibration was performed for the assessment of the inter- and intra-rater reliability of diagnosis. The dentists who were involved in the examination had been trained by Japanese dentists, including the two authors (TN and AA), through a previous project that was implemented in two primary schools in the study district between 2008 and 2011.

#### Child oral health behavior

The child oral health behavior examined in this study included the frequency of tooth brushing, visiting the dentist, and consuming sugary snacks/drinks daily. The frequency of tooth brushing was measured by the question “How many times does your child brush teeth a day?” with the response options of “Seldom or no brushing”, “Once”, “Twice”, and “Three times or more”. The frequency of visiting the dentist was measured by the question “How often does your child visit a dentist?” with the response options of “Regularly every 6–12 months”, “Occasionally”, “When he/she had a dental pain”, and “He/she never visited”. The frequency of consuming sugary snacks/drinks daily was measured by the question “Does your child take sugary snacks/drinks on a daily basis?” with the response options of “Yes”, “No”, and “Not sure/I don’t remember”.

#### Guardian’s beliefs

The guardian’s beliefs related to the following five domains were examined according to the HBM: perceived susceptibility of her/his child to dental caries, perceived severity of her/his child’s dental caries, perceived benefit of her/his child’s tooth brushing, perceived barrier to her/his child’s tooth brushing, and self-efficacy in making her/his child brush their teeth twice per day. These beliefs were assessed using a question about how much the respondent agreed with a statement. For perceived susceptibility, the statement was “My child will develop tooth decay next year”. For perceived severity, the statement was “If my child gets tooth decay, it is very serious”. For perceived benefit, the statement was “If my child brushes his/her teeth properly, he/she can prevent tooth decay”. For perceived barrier, the statement was “My child does not like brushing teeth”. For self-efficacy, the statement was “I am confident that I can make my child brush his/her teeth twice per day”. These question items were based on previous studies [13,14]. A five-point Likert scale was used for the response choice, ranging from “Strongly disagree” to “Strongly agree”. Except for the perceived barrier, which had a reverse scoring statement, five points were given for “Strongly agree” and one point was given for “Strongly disagree”.

#### Socio-demographic and economic characteristics

For guardians, the socio-demographic characteristics examined in this study included age, gender, relationship to the child, and occupation. For children, the socio-demographic characteristics were gender and school grade (as a proxy measure of age). Household economic status was measured by an asset-based wealth index, which has been validated elsewhere and has been widely used in developing countries [16]. Guardians were asked to indicate whether their households possess the following assets: TV, PC, DVD player, fixed phone, refrigerator, and car/truck. The principal component analysis was used to assess the weight of these household assets and to build an asset index (a proxy for economic status) by which households were divided into quartiles. As a result of the analysis, two principal components were extracted and the first component, which explained 33.0% of the total variance, was used for the asset index. The percentage of households that possessed the asset (scoring factor of the asset) was 93.1% (0.550) for television, 58.5% (0.638) for DVD player, 24.3% (0.582) for personal computer, 39.3% (0.481) for fixed phone, 90.4% (0.592) for refrigerator and 29.8% (0.594) for car/truck.

### Statistical analysis

Logistic regression analysis was conducted to assess the association between child dental caries status and child oral health behavior, adjusting for possible confounders such as sex and school grade. Logistic regression analysis was also conducted to assess the association between child tooth-brushing behavior and the guardians’ beliefs, adjusting for possible confounders such as a relationship with the child and household economic status measured by the asset index. For the multivariate model that assessed the association between child tooth-brushing behavior and the guardians’ beliefs, the pseudo-R-squared value was computed to assess the model fit. A mixed-effects model was used to control for the clustering within schools. Chi-square trend analysis was conducted to assess the linear trend between the five levels of guardians’ beliefs, which ranged from “very low” to very high”, and the proportion of children who brushed their teeth twice or more per day. Statistical analyses were performed using Stata 12 (StataCorp LP, College Station, TX, USA).

### Ethics statement

The protocol of the study was reviewed and approved by the National Ethics Committee for Health Research, Ministry of Health, Lao PDR and by the Ethical Review Committee for Epidemiological Research, University of the Ryukyus, Japan (No. 201), before the study began. Written informed consent was obtained from the guardians of the schoolchildren prior to the survey.

## Results

### General characteristics and oral health behavior of the children

Of the 1161 children, 622 (53.6%) were girls (S2 File). The number of children in each grade did not differ greatly, ranging from 214 to 254. The most common frequency of brushing teeth per day was twice (n = 573, 49.4%), followed by once (n = 306, 26.4%). Regarding the frequency of visiting a dental clinic, 721 children (62.1%) had visited a dental clinic only when they experienced pain. Five hundred fifty-seven children (48.0%) consumed sugary snacks or drinks on a daily basis.

### General characteristics of the guardians

The 1161 guardians who participated in the survey were mostly either mothers (53.4%) or fathers (37.6%). The median age of the guardians was 37-years-old, with the interquartile range from 32- to 42-years-old. The most common occupation was factory worker (31.0%), followed by housewife (30.0%), public servant (19.9%), and salesperson (15.2%).

### Associations between dental caries and child behavior

The prevalence of dental caries was 82% (951/1161). The bivariate analysis showed that children who brushed their teeth twice or more per day were significantly less likely to have dental caries than those who did not (odds ratio [OR]: 0.58, 95% confidence interval [CI]: 0.42 to 0.81) (Table 1). Additionally, children who consumed sugary snacks or drinks on a daily basis were significantly more likely to have dental caries than those who did not (OR: 1.45, 95% CI: 1.07 to 1.96). These associations remained significant even after adjustment for other variables in the multivariate analysis.

**Table 1:** Associations between dental caries status and oral health behaviors.

Variables	Caries prevalence (%)	Bivariate analysis			Multivariate analysis^1^		
		OR	95% CI	*p*-value	OR	95% CI	*p*-value
**Brushing teeth twice or more often**							
No	86.6	1.00	Reference		1.00	Reference	
Yes	79.1	0.58	0.42 to 0.81	0.001	0.62	0.44 to 0.88	0.008
**Visiting dentist regularly**							
No	82.5	1.00	Reference		1.00	Reference	
Yes	76.9	0.70	0.45 to 1.11	0.129	0.74	0.45 to 1.22	0.244
**Consuming sugary snacks/drinks daily**							
No	79.3	1.00	Reference		1.00	Reference	
Yes/Not sure	84.7	1.45	1.07 to 1.96	0.017	1.42	1.02 to 1.96	0.033

^1^ Adjusted for brushing teeth twice or more a day, visiting dentist regularly, consuming sugary food/drink daily, gender and school grade

### Associations between tooth-brushing behavior of children and guardians’ beliefs

The bivariate analysis showed that the tooth-brushing behavior of the children was significantly associated with guardians’ self-efficacy (Table 2): Children whose guardians had high self-efficacy were significantly more likely to comply with tooth brushing twice or more daily than were children whose guardians had low self-efficacy (OR: 2.14, 95% CI: 1.91 to 2.41). The association remained significant even after adjustment for other variables in the multivariate analysis.

**Table 2:** Associations between tooth brushing behavior of children and oral health belief of guardians.

	Bivariate analysis			Multivariate analysis^1^		
Variables	OR	95% CI	*p*-value	OR	95% CI	*p*-value
**Susceptibility**	0.98	0.89 to 1.76	0.650	1.02	0.90 to 1.14	0.791
**Severity**	1.09	0.99 to 1.20	0.085	1.13	0.99 to 1.26	0.064
**Benefit**	1.08	0.97 to 1.21	0.167	1.04	0.91 to 1.20	0.533
**Barrier**	1.08	0.97 to 1.21	0.167	0.91	0.78 to 1.07	0.245
**Self-efficacy**	2.14	1.91 to 2.41	<0.001	2.24	1.97 to 2.54	<0.001

^1^ adjusted for susceptibility, severity, benefit, barrier, self-efficacy, relationship to child and asset index

In the full model, the pseudo-R-squared value was 0.146, indicating that 14.6% of the variance in children’s tooth-brushing behavior was explained by the guardians’ beliefs, the relationship between guardians and children, and asset index. In the model that included guardians’ belief variables alone, the pseudo-R-squared value was 0.136, indicating that 13.6% of the variance in children’s tooth-brushing behavior was explained by the guardians’ beliefs.

There was a significant linear relationship between children’ tooth brushing and guardians’ self-efficacy (*P* < 0.001, Table 3): The frequency of children who often complied with tooth brushing twice or more daily increased with the increasing level of guardians’ self-efficacy. For example, 28.2% of children whose guardian showed very low self-efficacy complied with brushing twice or more often daily, whereas 84.2% of children whose guardians showed very high efficacy complied with the same behavior.

**Table 3:** Cross tabulation between guardians’ belief and tooth brushing behavior of their children.

Guardians’ belief	Guardians whose child brushed teeth twice/more often (n = 727)		Guardians whose child brushed teeth once/less often (n = 434)		*P* for trend
	n	%	n	%	
**Perceived susceptibility to child dental caries**					
Very low	114	60.0	76	40.0	0.487
Low	118	74.2	41	25.8	
Neutral	240	59.1	166	40.9	
High	150	65.8	78	34.2	
Very high	90	58.4	64	41.6	
**Perceived severity to child dental caries**					
Very low	56	58.9	39	41.1	0.104
Low	57	62.6	34	37.4	
Neutral	35	60.3	23	39.7	
High	280	58.8	196	41.2	
Very high	287	67.5	138	32.5	
**Perceived benefit of child’s tooth brushing**					
Very low	23	47.9	25	52.1	0.176
Low	43	55.1	35	44.9	
Neutral	59	61.5	37	38.5	
High	337	66.9	167	33.1	
Very high	251	60.0	167	33.1	
**Perceived barrier to the child’s tooth brushing**					
Very low	15	48.4	16	51.6	0.392
Low	75	63.0	44	37.0	
Neutral	214	64.3	119	35.7	
High	321	60.7	208	39.3	
Very high	82	68.3	38	31.7	
**Self-efficacy in making child brush twice per day**					
Very low	24	28.2	61	71.8	<0.001
Low	126	43.6	163	56.4	
Neutral	104	47.5	115	52.5	
High	284	82.6	60	17.4	
Very high	176	84.2	33	15.8	

## Discussion

The present study showed that children who brushed their teeth twice or more per day were significantly less likely to have dental caries than children who brushed once per day or less often. This finding is reasonable because a previous systematic review with meta-analysis showed that there was a significant difference in the incidence of caries between frequent brushers (i.e., twice or more per day) and infrequent brushers (i.e., once or less than once per day) [17].

The present study also showed that guardians’ self-efficacy in making their children brush their teeth twice daily was significantly associated with the tooth-brushing behavior of their children. This finding is consistent with those of previous work. An international study involving 17 countries reported that parents’ perception of their ability to control their children’s tooth brushing was a strong predictor of the tooth-brushing and snacking behavior of their children aged 3 to 4 years [10]. Also, a study of primary school children in Australia reported that higher parental self-efficacy was associated with more frequent tooth brushing by parent and child [18]. Intervention studies suggested that parents’ self-efficacy was modifiable: an intervention using a storybook that was embedded with behavioral change techniques showed that parental self-efficacy and intention to enact oral health behavior for their young children was significantly improved in England [19]. Similarly, four small 90-min group sessions that provided educational information, direct instruction, practice, and peer-to-peer problem solving improved parental self-efficacy for tooth brushing, and the proportion of parents who reported brushing their young children’s teeth twice per day increased significantly in the USA [20]. Therefore, there is a possibility that an intervention aimed at improving the self-efficacy of parents would be effective in improving the brushing frequency of their children in the Lao PDR. We recommend that such interventions in the Lao PDR should also utilize a peer-to-peer problem-solving approach. In the present study 18% of the guardians had a very high level of self-efficacy in making their children brush their teeth twice per day. Such guardians could play a major role in sharing their problem-solving experience with other guardians. In many primary schools of the Lao PDR, guardians are supposed to gather at school at the beginning of every semester. Such existing opportunities could be utilized for the intervention.

The present study’s HBM might not be the best theoretical framework to explain schoolchildren’s tooth-brushing behavior by their guardians’ beliefs in an urban area of Lao PDR. This is because only one construct of the HBM (i.e., guardians’ self-efficacy) was found to be significantly associated with children’s tooth-brushing behavior. This is also because the model with HBM constructs alone explained only 13.6% of the variation in children’s tooth-brushing behavior. This suggests the presence of an important factor that was not included in the present study’s HBM that focused only on guardians’ beliefs. Children’s beliefs might be one such important factor [21].

A study that compared five cognitive theories with medical students in Romania found that Theory of Planned Behavior (TPB) better explained the variation in students’ tooth-brushing behavior than other four theories including HBM; 22.7% by TPB vs. 10.2% by HBM [22]. Therefore, there is a possibility that TPB is more appropriate than HBM in explaining tooth-brushing behavior. However, whether some theoretical framework better explains tooth-brushing behavior than others would depends, at least partly, on study settings [7].

In the present study, the children who consumed sugary snacks/drinks on a daily basis were significantly more likely to have dental caries. There is a wealth of evidence that shows the role of dietary sugars in the etiology of dental caries [23]. In the present study, 48% of children consumed sugary snacks/drinks daily. This percentage is not surprisingly high. A Lao study that was conducted with 12-year-old children in the Vientiane capital in 2006 showed that 31% consumed sweets daily and 48% consumed sugary drinks frequently at school [2]. The 17-country international study also reported that parents’ perception of their ability to control their children’s sugar snacking was a significant predictor of the snacking behavior of their children aged 3 to 4 years [10]. Therefore, it might be possible for children in the Lao PDR to reduce the consumption of sugary snacks and drinks if their parents modify their self-efficacy for reducing their children’s consumption of these items.

The consumption of sugary snacks and drinks is influenced by factors external to the family including the school environment [3, 24]. In urban areas of the Lao PDR, sugary foods and drinks are easily available and affordable. A health promotion approach that restricts the availability of sugary snacks/drinks at school is one potential approach to reducing their consumption [25].

This study has several major limitations. First, because this was not a prospective study, it was unable to ascertain the causality of the associations found. Second, because the project was in place in the study schools, results might have been affected by the activities of the project. The prevalence of dental caries could be lower in the study schools than in other schools in urban areas of the Lao PDR. Additionally, children and their guardians in the study schools might have been more aware of oral health. Third, although there were 40 primary schools (20 public and 20 private schools) in the district, the study schools were the 10 public primary schools that were purposively selected from the 20 public schools. Therefore, the results of the present study could not represent the schoolchildren and their guardians in the district. Fourth, because no calibration was performed in the present study, no information is available about the degree of agreement on diagnosis between and within the dentists. The lack of calibration might have compromised the finding of the present study. Fifth, the HBM instrument used in the present study was not previously validated by means of robust analysis. The fact might have compromised the finding of the present study. Finally, the first principal component explains only 33% of the variation in the six assets variables, although this proportion could be considered substantial [26].

## Conclusions

The presence of childhood dental caries was significantly associated both with twice or more daily tooth brushing and with the daily consumption of sugary snacks/drinks. Children’s tooth-brushing behavior was significantly associated with guardians’ self-efficacy in their ability to make their children brush their teeth.

## Supporting information

S1 File Questionnaire. (DOC)

S2 File Table: Characteristics and oral health behavior of the children. (DOCX)

S3 File Dataset. (XLSX)

S4 File Editing certificate. (PDF)
