# Supplementary material for: Oral health behavior of children and guardians’ beliefs about children’s dental caries in Vientiane, Lao People’s Democratic Republic (Lao PDR)
Source: PLoS One. 2019 Jan 25;14(1):e0211257. doi: 10.1371/journal.pone.0211257 (PMC6347166; doi:10.1371/journal.pone.0211257)
Supplement: S1 File — (DOC) [file pone.0211257.s001.doc]

**Questionnaire**

**How to fill this questionnaire**

- Write down your answer on the spaces highlighted in gray color.

*Example*: Name of the student: Mr. ○○○○○○○○○○

- Choose only one answer and check the box.

*Example*: Is your child covered by health insurance?

☐^1^ Yes ☑^2^ No ☐^3^ Not sure/I don’t know

**Name of the student:**

**Name of the school to which the student goes:**

**Class/grade of the student:**

**Your name:**

**Your age:**

**Your gender:** ☐Male　☐Female ☐Other

**Your relationship to the student:**

**Your occupation:**

**Household assets**

Please tick the box ☑ if your household has.

☐Television ☐VCD/DVD player

☐Non-mobile phone (fixed phone) ☐Computer

☐Refrigerator ☐Domestic animals (chicken, duck, pig, cow, etc.)

☐Car/Truck ☐Rice field/farm

1. Is your child covered by health insurance?

☐^1^ Yes ☐^2^ No ☐^3^ Not sure/I don’t know

2.My child has now tooth decay.

☐^1^Strongly disagree ☐^2^Disagree ☐^3^Not sure ☐^4^Agree ☐^5^Strongly agree

3. My child will develop tooth decay next year.

☐^1^Strongly disagree ☐^2^Disagree ☐^3^Not sure ☐^4^Agree ☐^5^Strongly agree

4. If my child gets tooth decay, it is very serious.

☐^1^Strongly disagree ☐^2^Disagree ☐^3^Not sure ☐^4^Agree ☐^5^Strongly agree

5. If my child gets tooth decay, he/she suffers from severe pain.

☐^1^Strongly disagree ☐^2^Disagree ☐^3^Not sure ☐^4^Agree ☐^5^Strongly agree

6. If my child gets tooth decay, he/she loses his/her teeth.

☐^1^Strongly disagree ☐^2^Disagree ☐^3^Not sure ☐^4^Agree ☐^5^Strongly agree

7. If my child gets tooth decay, it affects his/her overall health.

☐^1^Strongly disagree ☐^2^Disagree ☐^3^Not sure ☐^4^Agree ☐^5^Strongly agree

8. If my child brushes his/her teeth properly, he/she can prevent tooth decay.

☐^1^Strongly disagree ☐^2^Disagree ☐^3^Not sure ☐^4^Agree ☐^5^Strongly agree

9. If my child brushes teeth properly, his/her teeth will remain in his/her lifetime.

☐^1^Strongly disagree ☐^2^Disagree ☐^3^Not sure ☐^4^Agree ☐^5^Strongly agree

10. My child does not like brushing teeth.

☐^1^Strongly disagree ☐^2^Disagree ☐^3^Not sure ☐^4^Agree ☐^5^Strongly agree

11. Toothbrush is expensive.

☐^1^Strongly disagree ☐^2^Disagree ☐^3^Not sure ☐^4^Agree ☐^5^Strongly agree

12. Toothpaste is expensive.

☐^1^Strongly disagree ☐^2^Disagree ☐^3^Not sure ☐^4^Agree ☐^5^Strongly agree

13. My child does not like the taste of toothpaste.

☐^1^Strongly disagree ☐^2^Disagree ☐^3^Not sure ☐^4^Agree ☐^5^Strongly agree

14. I am confident that I can make my child brush his/her teeth twice a day.

☐^1^Strongly disagree ☐^2^Disagree ☐^3^Not sure ☐^4^Agree ☐^5^Strongly agree

15. I am confident that I can make my child take sugary snacks/drinks in moderation.

☐^1^Strongly disagree ☐^2^Disagree ☐^3^Not sure ☐^4^Agree ☐^5^Strongly agree

16. I am confident that I can teach my child oral hygiene care properly.

☐^1^Strongly disagree ☐^2^Disagree ☐^3^Not sure ☐^4^Agree ☐^5^Strongly agree

17. How many times does your child brush teeth a day?

☐^1^ Seldom or no brushing ☐^2^ Once ☐^3^ Twice ☐^4^ Three times or more

18. Does your child brush teeth after dinner?

☐^1^ Yes ☐^2^ No ☐^3^ Not sure/I don’t know

19. How long does it take for your child to brush teeth each time?

☐^1^ Less than 1 minute ☐^2^ One minute ☐^3^ Two minutes ☐^4^ Three minutes or longer

20. How often do you replace a toothbrush of your child?

☐^1^ Every month or more often ☐^2^ Every 2-3 months ☐^3^ Every 4-5 months ☐^4^ Every 6 months or less often

21. Does your child use dental floss?

☐^1^Yes, he/she uses ☐^2^No, he/she doesn’t use ☐^3^Not sure/I don’t know

22. Have you ever provided your child with the final brushing?

☐^1^Yes, I have ☐^2^No, I haven’t ☐^3^Not sure/I don’t know

23. In the last week, did you provide your child with the final brushing?

☐^1^Yes, I did ☐^2^No, I didn’t ☐^3^Not sure/I don’t know

24. Have you ever taught your child tooth brushing techniques?

☐^1^Yes, I have ☐^2^No, I haven’t ☐^3^Not sure/I don’t know

25. Have you ever checked the teeth of your child after his/her brushing?

☐^1^Yes, I have ☐^2^No, I haven’t ☐^3^Not sure/I don’t know

26. In the last week, did you check the teeth of your child after his/her brushing?

☐^1^Yes, I did ☐^2^No, I didn’t ☐^3^Not sure/I don’t know

27. How often does your child visit a dentist?

☐^1^Regularly every 6-12 months ☐^2^Occasionally

☐^3^When he/she had a dental pain ☐^4^He/she never visited

28. Last time, when did your child visit a dentist for dental treatment?

Year: Month:

29. Last time, where did your child visit for dental treatment?

☐^1^Dental clinic ☐^2^Setthathirath Hospital

☐^3^District Hospital ☐^4^Other

30. Did your child visit a dentist when he/she had a dental pain?

☐^1^Yes ☐^2^No ☐^3^Not sure/I don’t remember

31. If you answer “No” at the question above, select the main reason for not visiting a dentist.

☐^1^My child feared dentist very much ☐^2^No money for treatment

☐^3^No time to take my child to dentist ☐^4^No hospital/dental clinic near my house

☐^5^Other

32. Do you tell your child not to take sugary snacks/drinks a lot?

☐^1^Yes ☐^2^No ☐^3^Not sure/I don’t remember

33. Does your child take sugary snacks/drinks on a daily basis?

☐^1^Yes ☐^2^No ☐^3^Not sure/I don’t remember

Thank you very much for your cooperation.
