# Supplementary material for: Oral health behavior of children and guardians’ beliefs about children’s dental caries in Vientiane, Lao People’s Democratic Republic (Lao PDR)
Source: PLoS One. 2019 Jan 25;14(1):e0211257. doi: 10.1371/journal.pone.0211257 (PMC6347166; doi:10.1371/journal.pone.0211257)
Supplement: S2 File — (DOCX) [file pone.0211257.s002.docx]

**Table: Characteristics and oral health behavior of the children**

| **Characteristics and behavior** | **n (n=1161)** | **%** |
| --- | --- | --- |
| **Sex** |  |  |
| Female | 622 | 53.6 |
| Male | 539 | 46.4 |
| **School grade** |  |  |
| First (around 6 to 7 years old) | 224 | 19.3 |
| Second (around 7 to 8 years old) | 222 | 19.1 |
| Third (around 8 to 9 years old) | 247 | 21.3 |
| Fourth (around 9 to 10 years old) | 254 | 21.9 |
| Fifth (around 10 to 11 years old) | 214 | 18.4 |
| **The frequency of brushing teeth per day** |  |  |
| Seldom or no | 128 | 11.0 |
| Once | 306 | 26.4 |
| Twice | 573 | 49.4 |
| Three times or more | 154 | 13.3 |
| **Visiting dental clinic** |  |  |
| Regularly every 6-12 months | 121 | 10.4 |
| Occasionally/when dental pain present | 721 | 62.1 |
| Have never visited | 319 | 27.5 |
| **Consuming sugary food/drink on a daily basis** |  |  |
| Yes | 557 | 48.0 |
| No | 259 | 22.3 |
| Not sure | 345 | 29.7 |
