# Supplementary material for: Oral health behavior of children and guardians’ beliefs about children’s dental caries in Vientiane, Lao People’s Democratic Republic (Lao PDR)
Source: PLoS One. 2019 Jan 25;14(1):e0211257. doi: 10.1371/journal.pone.0211257 (PMC6347166; doi:10.1371/journal.pone.0211257)
Supplement: S4 File — (PDF) [file pone.0211257.s004.pdf]

American Manuscript Editors

English Editing Certificate

This document certifies that the manuscript listed below was edited for proper English language, grammar, punctuation, and spelling by the expert staff at American Manuscript Editors.

Manuscript Title:

Oral health behavior of children and guardians' beliefs about children's dental caries in Vientiane, Lao People's Democratic Rep

Authors:

S. Phanthavong, D. Nonaka, T. Phonaphone, K. Kanda, P. Sombouaphan, N. Wake, S. Sayavong, T. Nakasone, K. Phongsavath, and A. Arasaki

Certificate Verification Key:

361-890-819-146-455

Project Number:

50357

This certificate may be verified by emailing info@americanmanuscripteditors.com. Documents receiving this certificate should be prepared for publication. However, please note that the author has the ability to accept or reject our suggestions for changes and can make changes after the editing process is complete, all of which can adversely affect the quality of the text after the editing process.

AMERICAN MANUSCRIPT EDITORS
